# Acetylcholine signaling genes are required for cocaine-stimulated egg laying in *Caenorhabditis elegans*

**DOI:** 10.1093/g3journal/jkab143

**Published:** 2021-04-29

**Authors:** Soren Emerson, Megan Hay, Mark Smith, Ricky Granger, David Blauch, Nicole Snyder, Rachid El Bejjani

**Affiliations:** 1 Neuroscience Interdisciplinary Program, Davidson College, Davidson, NC 28035, USA; 2 Biology Department, Davidson College, Davidson, NC 28035, USA; 3 Psychology Department, Davidson College, Davidson, NC 28035, USA; 4 Chemistry Department, Davidson College, Davidson, NC 28035 USA

**Keywords:** cocaine, *Caenorhabditis elegans*, egg laying, acetylcholine, serotonin, dopamine, tyramine, octopamine, acetylcholinesterase, L-amino acid decarboxylase, tyrosine beta-hydroxylase, tyrosine decarboxylase, nicotinic acetylcholine receptor, muscarinic acetylcholine receptor, vesicular acetylcholine transporter, Gq alpha subunit

## Abstract

The toxicity and addictive liability associated with cocaine abuse are well-known. However, its mode of action is not completely understood, and effective pharmacotherapeutic interventions remain elusive. The cholinergic effects of cocaine on acetylcholine receptors, synthetic enzymes, and degradative enzymes have been the focus of relatively little empirical investigation. Due to its genetic tractability and anatomical simplicity, the egg laying circuit of the hermaphroditic nematode, *Caenorhabditis elegans*, is a powerful model system to precisely examine the genetic and molecular targets of cocaine *in vivo*. Here, we report a novel cocaine-induced behavioral phenotype in *C. elegans*, cocaine-stimulated egg laying. In addition, we present the results of an *in vivo* candidate suppression screen of synthetic enzymes, receptors, degradative enzymes, and downstream components of the intracellular signaling cascades of the main neurotransmitter systems that control *C. elegans* egg laying. Our results show that cocaine-stimulated egg laying is dependent on acetylcholine synthesis and synaptic release, functional nicotinic acetylcholine receptors, and the *C. elegans* acetylcholinesterases.

## Introduction

Cocaine abuse is estimated to account for 900,000 cases of substance use disorder and over 14,000 overdose deaths in the United States each year (Lipari and Van Horn 2013; Hedegaard *et al.* 2020). Despite the impact of cocaine abuse on public health, there are no FDA-approved pharmacotherapeutic interventions for either the addictive liability or toxicity associated with cocaine abuse (Kampman 2019). A major impediment to the development of effective pharmacological interventions is the nonspecificity of the drug, as pharmacodynamic interventions designed to block the canonical monoamine neurotransmitters implicated in the mode of action of cocaine [dopamine (DA), serotonin (5-HT), and norepinephrine] have demonstrated limited efficacy in clinical trials (Pitts and Marwah 1989; Czoty *et al.* 2016). Elucidation of the full range of neurotransmitter systems and molecular effectors involved in the mode of action of cocaine could inform the development of novel pharmacotherapies.

Cholinergic signaling influences multiple processes underlying reward and dependence in the mammalian brain, including learning, attention, and motivation (Hasselmo 2006; Sofuoglu and Mooney 2009; Klinkenberg *et al.* 2011). In addition, behavioral and physiological research demonstrates an important role for acetylcholine (ACh) in the pathology of cocaine use disorder (Williams and Adinoff 2008). However, the cholinergic mechanisms of cocaine have not been fully investigated.

The *Caenorhabditis elegans* egg laying circuit has served as a tractable genetic and molecular model for the mechanistic underpinnings of various neurotransmitters, including ACh and the biogenic amines 5-HT, DA, octopamine (Oct), and tyramine (Tyr) (Schafer 2005; Chase 2007). The circuit consists of two hermaphrodite-specific motor neurons (HSNs) and six ventral type C motor neurons (VCs) that innervate eight vulval muscles (Schafer 2006). ACh plays a crucial and complex role in regulating the egg laying behavior of *C. elegans* (Rand 2007). The VCs, which are the primary cholinergic neurons in the egg laying circuit, innervate both the vulval muscles and the HSNs (White *et al.* 1986). Although typically regarded as serotonergic motor neurons, the HSNs have also been reported to function as cholinergic motor neurons in the egg laying circuit (Duerr *et al.* 2001; Pereira *et al.* 2015). ACh elicits excitatory and inhibitory effects on egg laying. The excitatory effect of ACh on egg laying is mediated by binding to nicotinic ACh receptors (nAChRs) on the vulval muscles inducing contraction and egg laying (Kim *et al.* 2001). Cholinergic inhibition of egg laying may result from binding of ACh to muscarinic ACh receptors (mAChRs) expressed in the egg laying circuit (Bany *et al.* 2003; Fernandez *et al.* 2020).

Here, we characterize a novel cocaine-induced behavioral phenotype in *C. elegans*, cocaine-stimulated egg laying, and present pharmacological and genetic evidence for the dependence of this behavioral phenotype on ACh, nAChRs, and acetylcholinesterases (AChEs). We show that the excitatory effect of cocaine on egg laying in *C. elegans* depends on genes involved in nicotinic cholinergic neurotransmission but not on the majority of genes encoding the molecular components of the other neurotransmitter systems expressed in the egg laying circuit. Together, our results provide a background for future investigation utilizing the *C. elegans* egg laying circuit to elucidate the cholinergic effects of cocaine.

## Materials and methods

### Nematode strains

The wild-type (WT) strain was Bristol N2. Animals were maintained on standard nematode growth media agar plates spread with *Escherichia coli* as a source of food (Stiernagle 2006). Temperature was controlled at 20°C. Supplementary Table S3 contains a complete list of all of the mutant *C. elegans* strains used in this study.

### Egg laying assays and statistical analysis

Egg laying assays in liquid media were performed according to a protocol by Moresco and Koelle (Hart 2006). For each experiment, an aqueous stock solution of 147.15 mM cocaine hydrochloride (50.000 mg/mL) was diluted to the desired treatment concentration in M9 buffer solution with an equal volume of 147.15 mM aqueous sucrose solution (50.369 mg/mL) diluted in M9 buffer solution in each of the control conditions. Late stage L4 hermaphrodites of the respective strains were picked ∼24 h before assaying and continued to be cultured on agar plates with an OP50 lawn at 20°C. Ten days one adult hermaphrodites were isolated into single wells on a 96-well plate containing 35 µl experimental or equimolar control solutions for each replicate. After 1 h, each well was examined, and the number of eggs laid by each animal was recorded.

For all liquid media egg laying assays, the investigators were blind to both the identity of the treatment and the genotype. Each experiment included 10 animals per experimental or control group, and each experiment was independently repeated five times. Seventeen total worms ruptured during experimental analysis. In these rare cases, the nonruptured worms in the replicate were used to score the egg laying response.

For the analysis of the mean egg laying response, the mean number of eggs laid by each of the five replicate treatment groups were treated as a single data point. For the analysis of fraction responding, defined as the fraction of animals laying greater than or equal to one egg, the fraction responding of each of the five replicate treatment groups was treated as a single data point. Statistical analysis of the mean egg laying response and the fraction responding was performed using a Mann–Whitney *U*-test.

### Chemicals

Aqueous cocaine hydrochloride was supplied by the National Institute on Drug Abuse (Research Triangle Institute, Research Triangle Park, NC, USA).

### Data availability

All mutant strains examined in this study are available to order from the *Caenorhabditis* Genetics Center, which is funded by the National Institutes of Health Office of Research Infrastructure Programs (P40 OD010440). Some strains were generated by the *C. elegans* Deletion Mutant Consortium. The authors affirm that all data necessary for confirming the conclusions of this article are represented fully within the article and its tables and figures. Supplemental Material available at figshare: https://doi.org/10.25387/g3.14477901.

## Results

### High concentrations of cocaine overcome the inhibition of egg laying in hypertonic liquid media

**Figure 1:**
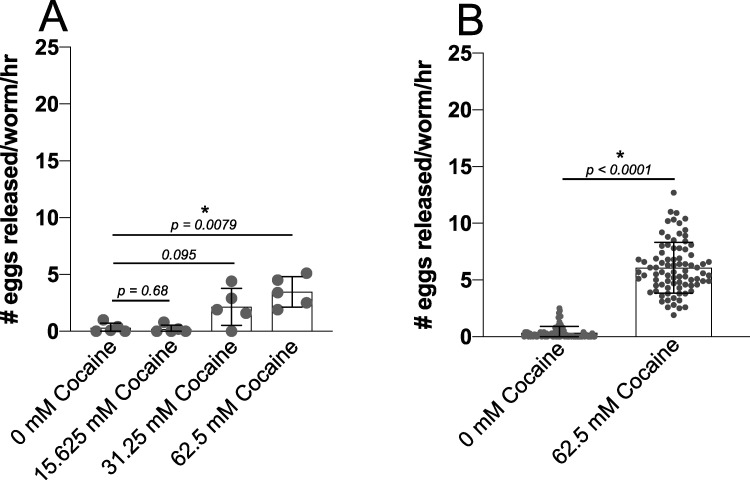
Cocaine stimulates *C. elegans* egg laying behavior. (A) Egg laying response of *C. elegans* to a series of increasing concentrations of cocaine in liquid media. (B) Egg laying response of *C. elegans* to 62.5 mM cocaine in liquid media compared to 62.5 mM sucrose control. For the data shown in (A), we performed five independent experiments with 10 animals in each group. The data shown in panel (B) show a compiled experiment for WT worms from our experiments (83 independent experiments with 10 animals in each group). Error bars represent SD. Mann–Whitney *U*-test. Significance indicated by asterisks.

In an effort to add to the current cocaine abuse models available in *C. elegans*, we quantified any differences in egg laying in *C. elegans* treated with cocaine by assaying the mean egg laying response over a time period of 1 h in hypertonic liquid media, a well-established method previously used to investigate the stimulation of egg laying by pharmacological agents (Horvitz *et al.* 1982; Trent *et al.* 1983; Desai and Horvitz 1989; Schafer and Kenyon 1995; Weinshenker *et al.* 1995; Jayanthi *et al.* 1998; Dempsey *et al.* 2005; Hart 2006; Ward *et al.* 2009; Engleman *et al.* 2016, 2018). We tested the mean egg laying response of *C. elegans* to a series of increasing concentrations of cocaine (0, 15.625, 31.25, and 62.5 mM) in liquid media for 1 h (Figure 1A). We show that treatment of WT *C. elegans* with 62.5 mM cocaine significantly stimulates egg laying compared to equimolar sucrose control [WT, 62.5 mM cocaine mean = 6.1 ± 2.2; WT, 62.5 mM sucrose control mean = 0.34 ± 0.56; *P *<* *0.0001, *N* = 83 replicates (10 animals/replicate)] (Figure 1B).

We used a 62.5 mM cocaine treatment and 62.5 mM sucrose control in all mutant analyses described in this paper. Interestingly, the concertation of cocaine necessary to robustly stimulate *C. elegans* egg laying behavior is greater than the concentrations of cocaine previously shown to modulate *C. elegans* locomotion by Ward *et al* (2009).

### ACh is the only neurotransmitter required for the stimulation of egg laying by cocaine in hypertonic liquid media

**Figure 2:**
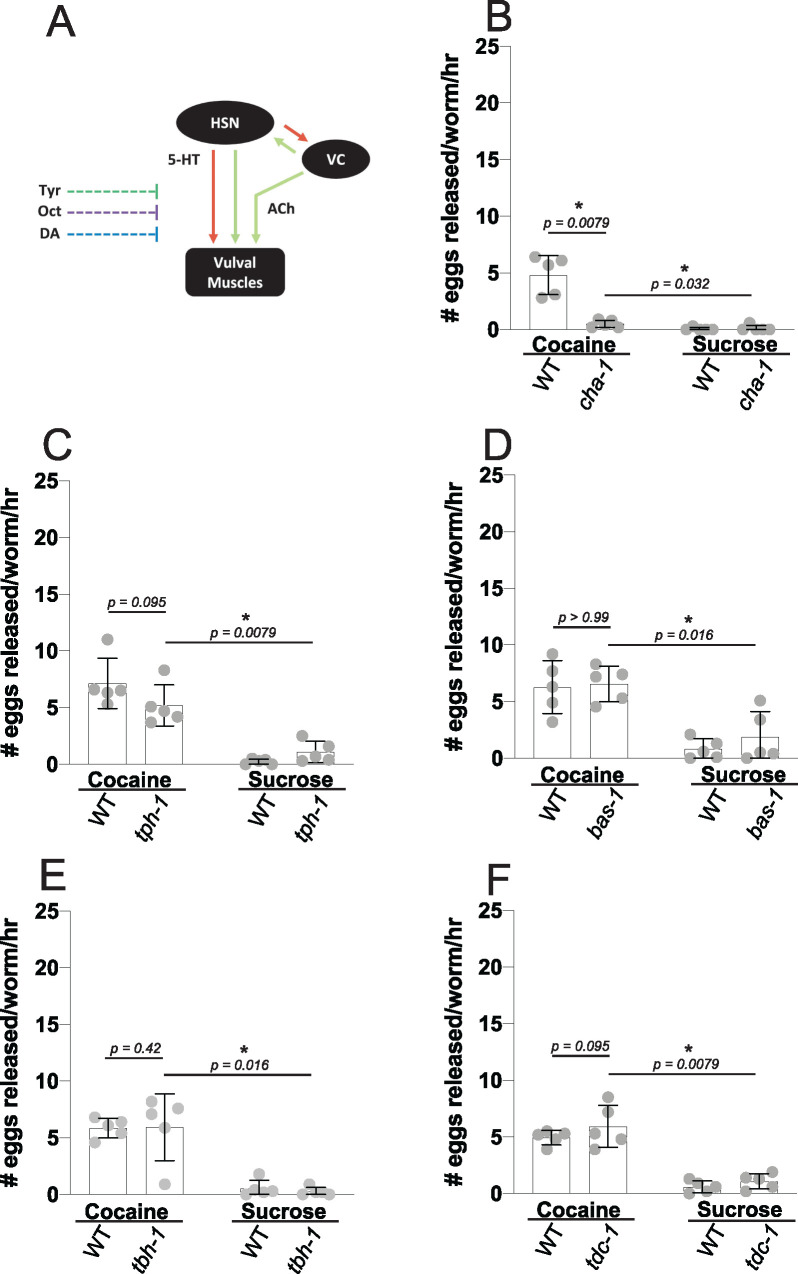
ChAT is required for the egg laying response to cocaine. (A) Schematic of the *C. elegans* egg laying circuit showing HSNs, VCs, the vulval muscles, and the neurotransmitter systems expressed in the egg laying circuit, which includes Tyr (dark green), Oct (purple), DA (blue), 5-HT (red), and ACh (light green). Extrasynaptic neurotransmitter systems marked with dashed lines. Synaptic neurotransmitter systems marked with solid lines. Egg laying behavior of mutants for (B). ChAT/*cha-1(p1152)*, (C). TPH/*tph-1(mg280)*, (D). AADC/*bas-1(ad446)*, (E). TDC*/tdc-1(n3419)*, and (F). TBH*/tbh-1(n3247)* in 62.5 mM cocaine or a 62.5 mM sucrose control compared to WT. We performed five independent experiments with 10 animals in each group. Error bars represent SD. Mann–Whitney *U*-test. Significance indicated by asterisks.

To begin investigating the neurobiological mechanism of cocaine-stimulated egg laying, we screened the egg laying response of individual mutants with defects in the synthetic enzymes for each of the neurotransmitters previously implicated in egg laying (Figure 2A) (Schafer 2005). Interestingly, a reduction of function allele of *cha-1(p1152)*, which encodes choline acetyltransferase (ChAT) necessary for ACh synthesis, suppresses egg laying in response to cocaine compared to WT [*cha-1(p1152)*, cocaine mean = 0.5 ± 0.3; WT, cocaine mean = 4.8 ± 1.8; *P *=* *0.0079, *N* = 5 replicates (10 animals/replicate)] (Figure 2B) (Rand and Russell 1984, 1985).

5-HT plays a well-described excitatory role in the egg laying circuit, and 5-HT signaling is one of the canonical targets of cocaine in mammals (Higgins and Fletcher 2003; Schafer 2005, 2006; Chase 2007; Müller *et al.* 2007). Surprisingly, a putative null allele of the *C. elegans* tryptophan hydroxylase (TPH), the 5-HT synthetic enzyme, did not suppress cocaine-stimulated egg laying compared to WT [*tph-1(mg280)*, cocaine mean = 5.2 ± 1.8; WT, cocaine mean = 7.1 ± 2.2; *P *=* *0.095, *N* = 5 replicates (10 animals/replicate)] (Figure 2C) (Sze *et al.* 2000). These results suggest that 5-HT, one of the main modulators of egg laying behavior and a canonical cocaine effector in mammalian systems, is not a primary target of cocaine in this model system.

In addition, putative null mutants for aromatic L-amino acid decarboxylase (AADC)/*bas-1(ad446)*, which encodes a 5-HT and DA synthetic enzyme, tyrosine beta-hydroxylase (TBH)*/tbh-1(n3247)*, which encodes an Oct synthetic enzyme, and tyrosine decarboxylase (TDC)/*tdc-1(n3419)*, which encodes a Tyr and Oct synthetic enzyme, do not exhibit a significant difference in the egg laying response to cocaine compared to WT, suggesting that neither DA, Tyr, nor Oct are necessary for cocaine-stimulated egg laying (Figure 2, D–F) (Hare and Loer 2004; Alkema *et al.* 2005).

### Cocaine-stimulated egg laying requires genes involved presynaptic ACh signaling

**Figure 3:**
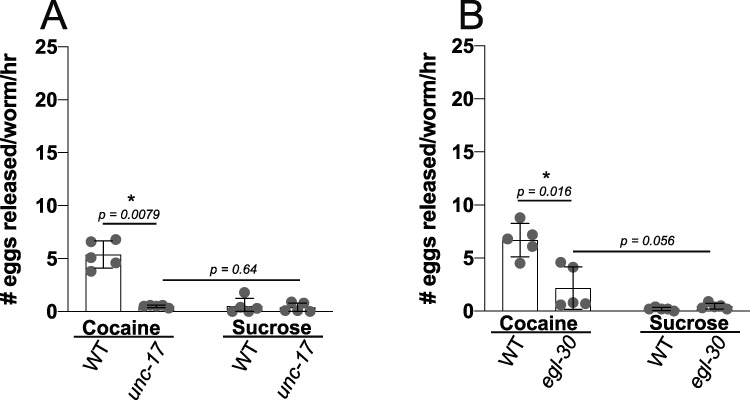
VAChT and Gα_q_ are required for the egg laying response to cocaine. Egg laying behavior of mutants for (A). VAChT/*unc-17(e245)* and (B) Gα_q_ *egl-30(ad806)*, which are required for ACh release from motor neurons, in 62.5 mM cocaine or a 62.5 mM sucrose control compared to WT. We performed five independent experiments with 10 animals in each group. Error bars represent SD. Mann–Whitney *U*-test. Significance indicated by asterisks.

Suppression of the egg laying response to cocaine in reduction of function ChAT/*cha-1(p1152)* mutants suggests that presynaptic cholinergic neurotransmission is necessary for the egg laying response to the drug. To further examine this possibility, we assayed cocaine-stimulated egg laying in mutants with a reduction of function mutation in the vesicular ACh transporter (VAChT)/*unc-17(e245)* necessary for packaging of ACh within synaptic vesicles (Alfonso *et al.* 1993). We show that the egg laying response of VAChT/*unc-17(e245)* mutants is suppressed compared to WT [*unc-17(e245)*, cocaine mean = 0.5 ± 0.1; WT, cocaine mean = 5.4 ± 1.3; *P *=* *0.0079, *N* = 5 replicates (10 animals/replicate)] and that cocaine does not stimulate a significant increase in egg laying in VAChT/*unc-17(e245)* mutants compared to equimolar sucrose control [*unc-17(e245)*, cocaine mean = 0.5 ± 0.1; *unc-17(e245)*, sucrose control mean = 0.4 ± 0.4; *P *=* *0.64, *N* = 5 replicates (10 animals/replicate)] (Figure 3A).

The Gα_q_ ortholog encoded by *egl-30* in *C. elegans* is required for ACh release from motor neurons, among other neuronal functions (Lackner *et al.* 1999). We show that mutants with a reduction of function mutation in Gα_q_/*egl-30(ad806)* exhibit suppressed cocaine-stimulated egg laying compared to WT [*egl-30(ad806)*, cocaine mean = 2.2 ± 2.0; WT, cocaine mean = 5.9 ± 0.8; *P *=* *0.016, *N* = 5 replicates (10 animals/replicate)] and that cocaine does not stimulate a significant increase in egg laying in Gα_q_/*egl-30(ad806)* mutants compared to equimolar sucrose control [*egl-30(ad806)*, cocaine mean = 2.2 ± 2.0; *egl-30(ad806)*, sucrose control mean = 0.5 ± 0.1; *P *=* *0.056, *N* = 5 replicates (10 animals/replicate)] (Figure 3B). Taken together, these results suggest that genes required for presynaptic ACh synthesis, packaging, and release are required for the egg laying response stimulated by cocaine in *C. elegans*.

### Genes encoding nAChRs, but not mAChRs, are required for the egg laying response to cocaine

**Figure 4:**
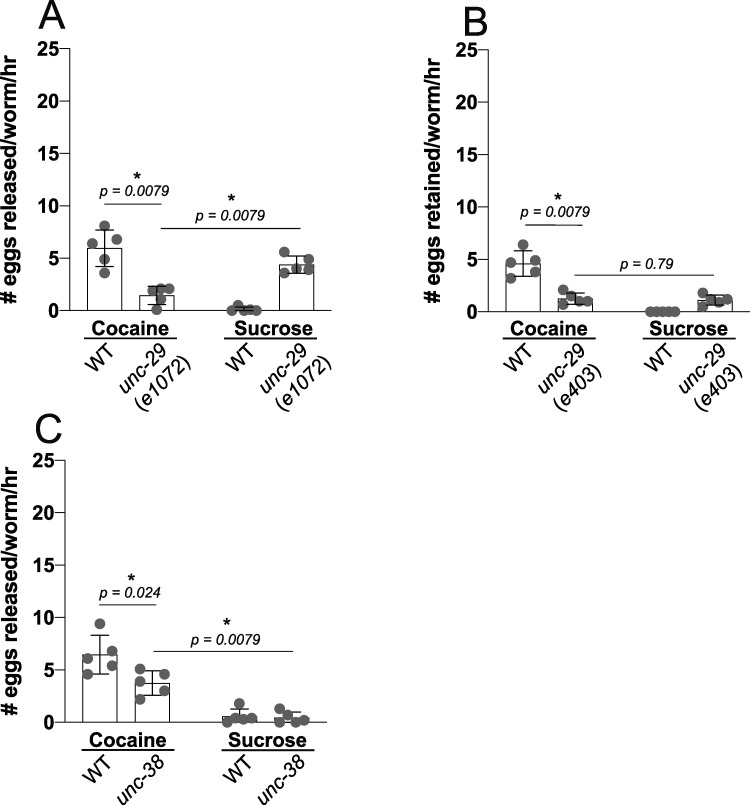
nAChRs are required for the egg laying response to cocaine. Egg laying behavior of (A) CHRNB/*unc-29(e1072)*, (B) CHRNB/*unc-29(e403)*, and (C). CHRNA/*unc-38(e264)* in 62.5 mM cocaine or a 62.5 mM sucrose control compared to WT. We performed five independent experiments with 10 animals in each group. Error bars represent SD. Mann–Whitney *U*-test. Significance indicated by asterisks.

**Figure 5:**
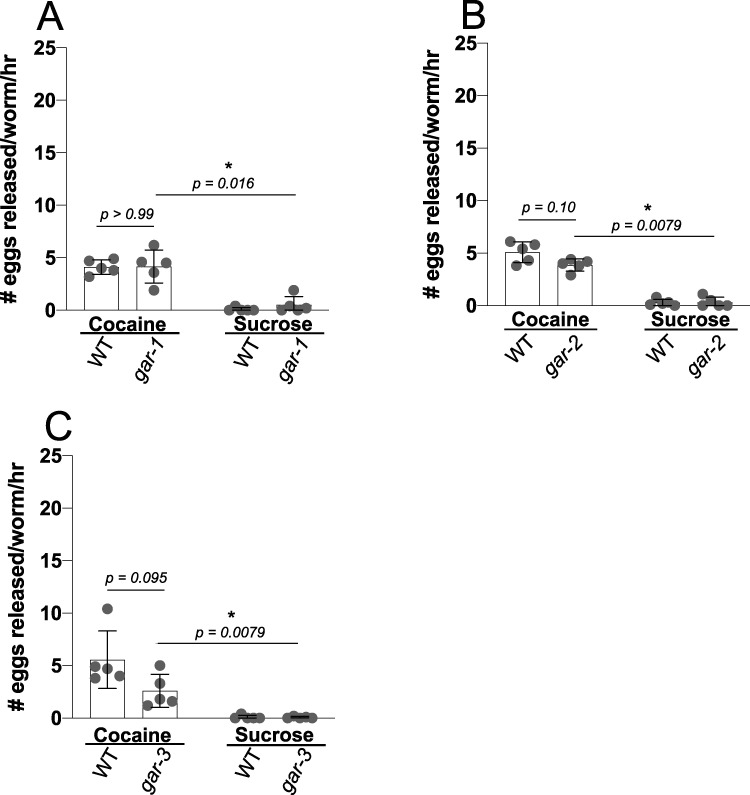
mAChRs are not required for the egg laying response to cocaine. Egg laying behavior of (A) *gar-1(ok755)*, (B) *gar-2(ok520)*, and (C) *gar-3(gk305)* in 62.5 mM cocaine or a 62.5 mM sucrose control compared to WT. We performed five independent experiments with 10 animals in each group. Error bars represent SD. Mann–Whitney *U*-test. Significance indicated by asterisks.

To further examine the effect of cocaine on cholinergic signaling in the *C. elegans* egg laying circuit, we tested the egg laying response to cocaine in nAChR and mAChR mutants (Figures 4 and 5) (Kim *et al.* 2001; Schafer 2006; Fernandez *et al.* 2020). Consistent with our finding that cholinergic neurotransmission is required for cocaine-stimulated egg laying, we show that the egg laying response to cocaine is significantly reduced compared to WT in *unc-29(e1072)* putative null mutants with defects in the beta subunit of nAChRs (CHRNB) [*unc-29(e1072)*, cocaine mean = 1.5 ± 0.97; WT, cocaine mean = 6.0 ± 1.7; *P *=* *0.0079, *N* = 5 replicates (10 animals/replicate)] (Figure 4A) (Fleming *et al.* 1997). Similarly, we show that the egg laying response to cocaine of alpha subunit nAChR putative null mutants (CHRNA)/*unc-38(e264)* is suppressed compared to WT [*unc-38(e264)*, cocaine mean = 3.8 ± 1.2; WT, cocaine mean = 6.5 ± 1.8; *P *=* *0.024, *N* = 5 replicates (10 animals/replicate)] (Figure 4C) (Richmond and Jorgensen 1999). These results suggest that nAChRs expressed on muscles, including the vulval muscles, are required for the molecular action of cocaine on *C. elegans* egg laying.

The response to cocaine of CHRNB/*unc-29(e1072)* mutants revealed an unexpected result. Specifically, although cocaine-stimulated egg laying is suppressed in CHRNB/*unc-29(e1072)* mutants compared to WT animals treated with cocaine, CHRNB/*unc-29(e1072)* mutants treated with cocaine show a significantly reduced egg laying response compared to that of CHRNB/*unc-29(e1072)* mutants treated with equimolar sucrose control [*unc-29(e1072)*, cocaine mean = 1.5 ± 0.97; *unc-29(e1072)* sucrose control mean = 4.4 ± 0.8; *P *=* *0.0079, *N* = 5 replicates (10 animals/replicate)] (Figure 4A). To determine if this effect is generalizable for other alleles of CHRNB/*unc-29*, we repeated the experiment in a second CHRNB/*unc-29(e403)* allele. We show that, as observed for the CHRNB/*unc-29(e1072)* allele, CHRNB/*unc-29(e403)* mutants also exhibit a suppressed egg laying response to cocaine compared to WT [*unc-29(e403)*, cocaine mean = 1.3 ± 0.6; WT, cocaine mean = 4.6 ± 1.2; *P *=* *0.0079, *N* = 5 replicates (10 animals/replicate)]. However, unlike CHRNB/*unc-29(e1072)*, the egg laying response to cocaine of CHRNB/*unc-29(e403)* mutants does not significantly differ from the egg laying response in CHRNB/*unc-29(e403)* treated with sucrose control [*unc-29(e403)*, cocaine mean = 1.3 ± 0.6; *unc-29(e403)*, sucrose control mean = 1.1 ± 0.5; *P *=* *0.79, *N* = 5 replicates (10 animals/replicate)] (Figure 4B). These results confirm the dependence of the egg laying response to cocaine on CHRNB/*unc-29* and suggest that the unexpected, reduced egg laying response to cocaine compared to sucrose control in CHRNB/*unc-29(e1072)* mutants may be allele-specific or due to an unidentified background mutation in this strain.

In addition to nAChRs, the *C. elegans* egg laying circuit expresses several mAChRs (Fernandez *et al.* 2020). To determine if mAChRs are required for the molecular mechanism of cocaine-stimulated egg laying, we assayed the egg laying response to cocaine of putative null mutants for the three mAChRs encoded by *gar-1(ok755)*, *gar-2(ok520)*, and *gar-3(gk305)* (Bany *et al.* 2003; Liu *et al.* 2007). We show all three mAChR mutants do not exhibit a significant difference in the egg laying response to cocaine compared to WT (Figure 5, A–C).

### AChE-encoding genes are required for the egg laying response to cocaine

**Figure 6:**
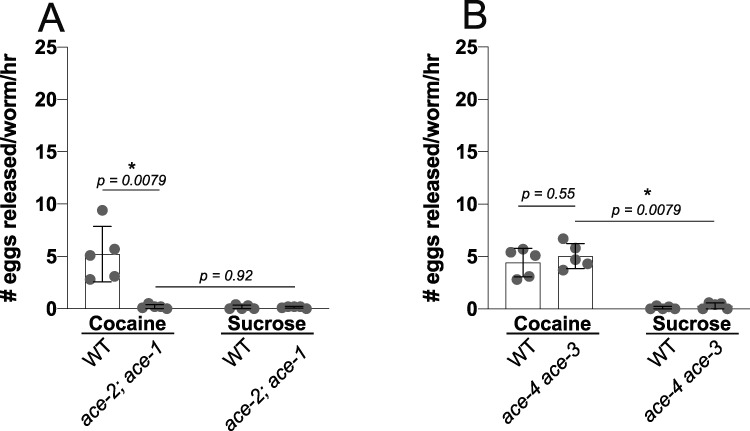
AChE-encoding genes are required for the egg laying response to cocaine. Egg laying behavior of (A) *ace-2(g72)*; *ace-1(p1000)* and (B) *ace-4 ace-3(dc2)* in 62.5 mM cocaine or a 62.5 mM sucrose control compared to WT. We performed five independent experiments with 10 animals in each group. Error bars represent SD. Mann–Whitney *U*-test. Significance indicated by asterisks.

AChEs catalyze the degradation of ACh at the synapse, decreasing cholinergic neurotransmission (Dvir *et al.* 2010). Biochemical analysis has revealed multiple genes encoding *C. elegans* AChEs, including *ace-1*, *ace-2*, *ace-3*, and *ace-4* (Combes *et al.* 2001). The AChEs encoded by *ace-1* and *ace-2* are the major degradative enzymes of ACh whereas the AChE encoded by *ace-3* accounts for a minor proportion of AChE activity, and the AChE encoded by *ace-4* is transcribed but does not result in a catalytically active protein (Johnson and Russell 1983; Kolson and Russell 1985; Arpagaus *et al.* 1998; Rand 2007). In order to explore all aspects of the relationship between cocaine and cholinergic signaling in the egg laying response, we tested the egg laying response to cocaine of *ace-2(g72)*; *ace-1(p1000);* and *ace-4 ace-3(dc2)* putative null double mutants (Culotti *et al.* 1981; Combes *et al.* 2000). *ace-2(g72)*; *ace-1(p1000)* double mutants exhibit a significant decrease in the egg laying response to cocaine compared to WT [*ace-2(g72)*; *ace-1(p1000)*, cocaine mean = 0.20 ± 0.19; WT, cocaine mean = 5.2 ± 2.6; *P *=* *0.0079, *N* = 5 replicates (10 animals/replicate)] (Figure 6A) whereas *ace-4 ace-3(dc2)* double mutants exhibit an egg laying response to cocaine that does not differ significantly compared to WT (Figure 6B).

### Cocaine-stimulated egg laying is dependent on the HSNs but not 5-HT receptor genes

**Figure 7:**
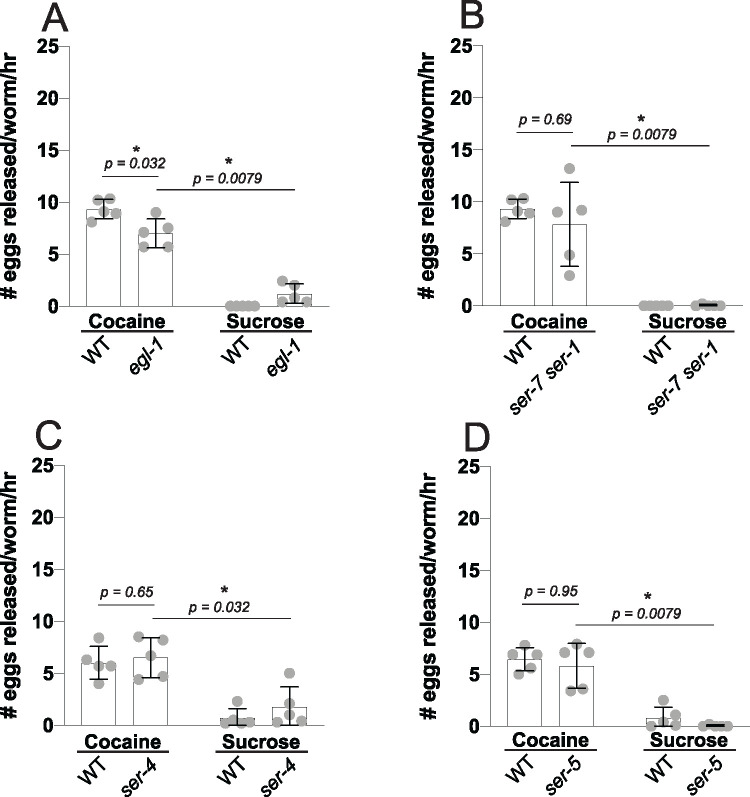
The HSNs, but not 5-HT receptors, are required for the egg laying response to cocaine. Egg laying behavior of (A) animals lacking HSNs**/***egl-1(n487)*, (B) *ser-7(tm1325) ser-1(ok345)* double mutants, (C) *ser-4(ok512)* mutants, and (D) *ser-5(ok3087)* mutants in 62.5 mM cocaine or a 62.5 mM sucrose control compared to WT. We performed five independent experiments with 10 animals in each group. Error bars represent SD. Mann–Whitney *U*-test. Significance indicated by asterisks.

The HSNs are the primary serotonergic motor neurons in the *C. elegans* egg laying circuit (Schafer 2006). In addition to their function as serotonergic motor neurons, the HSNs also function as cholinergic motor neurons (Duerr *et al.* 2001; Pereira *et al.* 2015). To further assess the role of the HSNs, 5-HT, and ACh in cocaine-stimulated egg laying, we quantified the egg laying response to cocaine in animals containing a semi-dominant allele of *egl-1*, shown to trigger HSN cell death (Conradt and Horvitz 1998). We show that *egl-1(n487)* mutants exhibit a slight but statistically significant difference in the egg laying response to cocaine compared to WT [*egl-1(n487)*, cocaine mean = 7.2 ± 1.2; WT, cocaine mean = 9.3 ± 0.9; *P *=* *0.032, *N* = 5 replicates (10 animals/replicate)] (Figure 7A). In addition, our data show that the HSNs contribute to cocaine-stimulated egg laying but that their presence is not necessary for the behavior. Specifically, cocaine stimulates a significant increase in egg laying compared to equimolar sucrose control in animals lacking the HSNs [*egl-1(n487)*, cocaine mean = 7.2 ± 1.2; *egl-1*(*n487*), sucrose control mean = 1.2 ± 0.9; *P *=* *0.0079, *N* = 5 replicates (10 animals/replicate)].

Our previous analysis of mutants with defects in cholinergic and serotonergic neurotransmission indicates that ACh, but not 5-HT, is required for the stimulatory effects of cocaine on *C. elegans* egg laying behavior. To determine if cocaine acts on 5-HT receptors in the egg laying circuit, we tested the egg laying response to cocaine in putative null mutants with defects in four metabotropic 5-HT receptors expressed in the *C. elegans* egg laying circuit. The genotypes tested include *ser-5(ok3087)*, which encodes an excitatory Gα_s_-coupled 5-HT receptor, *ser-4(ok512)*, which encodes an inhibitory Gα_o_-coupled 5-HT receptor, and a *ser-7(tm1325) ser-1(ok345)* double mutant, which encode an excitatory Gα_q_-coupled 5-HT receptor and an excitatory Gα_s_-coupled 5-HT receptor, respectively (Olde and McCombie 1997; Carnell *et al.* 2005; Hobson *et al.* 2006; Churgin *et al.* 2017). We show that *ser-7(tm1325) ser-1(ok345)* double mutants and *ser-5(ok3087)* mutants exhibit an egg laying response to cocaine that does not differ significantly compared to WT, suggesting that the excitatory post-synaptic effects of 5-HT are not required for cocaine-stimulated egg laying (Figure 7, B and D). Furthermore, our data suggest that cocaine does not stimulate egg laying by blocking the inhibitory effects of *ser-4(ok512)* (Figure 7C). Together, our data show that the egg laying response to cocaine depends on ACh receptors, but not 5-HT receptors, and suggest that suppression of the egg laying response to cocaine in *egl-1(n487)* mutants may result from a lack of presynaptic cholinergic neurotransmission by the HSN motor neurons.

### Cocaine-stimulated egg laying is increased in 5-HT-gated chloride channel/*mod-1* mutants and 5-HT reuptake transporter/*mod-5* mutants

**Figure 8:**
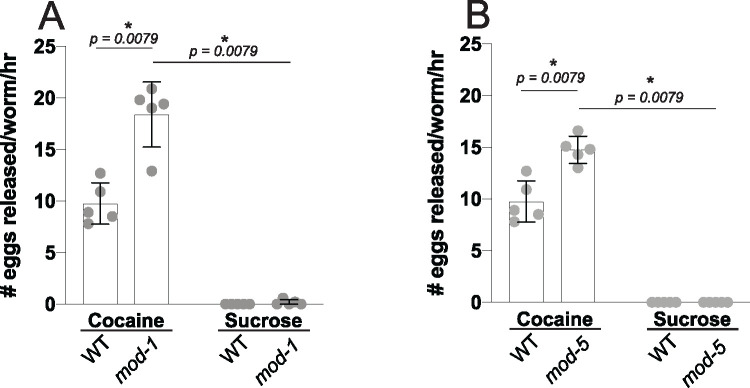
The mean egg laying response to cocaine is increased in 5-HT-gated chloride channel mutants and SERT mutants. (A) 5-HT-gated chloride channel/*mod-1(ok103)* mutants, (B) SERT/*mod-5(n3314)* mutants in 62.5 mM cocaine or a 62.5 mM sucrose control compared to WT. We performed five independent experiments with 10 animals in each group. Error bars represent SD. Mann–Whitney *U*-test. Significance indicated by asterisks.

Cocaine induces a decrease in locomotion speed in *C. elegans* that is dependent on the gene *mod-5*, which encodes the *C. elegans* 5-HT reuptake transporter (SERT), and the gene *mod-1*, which encodes an inhibitory 5-HT-gated chloride channel (Ward *et al.* 2009). We tested the egg laying response to cocaine in 5-HT-gated chloride channel/*mod-1(ok103)* putative null mutants and SERT/*mod-5(n3314)* putative null mutants to examine the role of these genes in cocaine-stimulated egg laying (Ranganathan *et al.* 2000; Ranganathan *et al.* 2001). 5-HT-gated chloride channel*/mod-1(ok103)* mutants exhibit a significant increase in the egg laying response to cocaine compared to WT [*mod-1(ok103)*, cocaine mean = 18 ± 3.2, WT, 62.5 mM cocaine mean = 9.8 ± 2.0; *P *=* *0.0079, *N* = 5 replicates (10 animals/replicate)] (Figure 8A). Similarly, SERT/*mod-5(n3314)* mutants exhibit a significant increase in the egg laying response to cocaine compared to WT [*mod-5(n3314)*, cocaine mean = 14.8 ± 1.3; WT, cocaine mean = 9.8 ± 2.0; *P *=* *0.0079, *N* = 5 replicates (10 animals/replicate)] (Figure 8B). The increased egg laying responses to cocaine in 5-HT-gated chloride channel*/mod-1(ok103)* mutants and SERT/*mod-5(n3314)* mutants suggest that the removal of 5-HT-gated chloride channel inhibition of egg laying or a global increase in 5-HT, respectively, may potentiate the egg laying response to cocaine.

### Cocaine-stimulated egg laying is weakly suppressed in DA reuptake transporter mutants but not mutants with defects in other DA signaling genes

**Figure 9:**
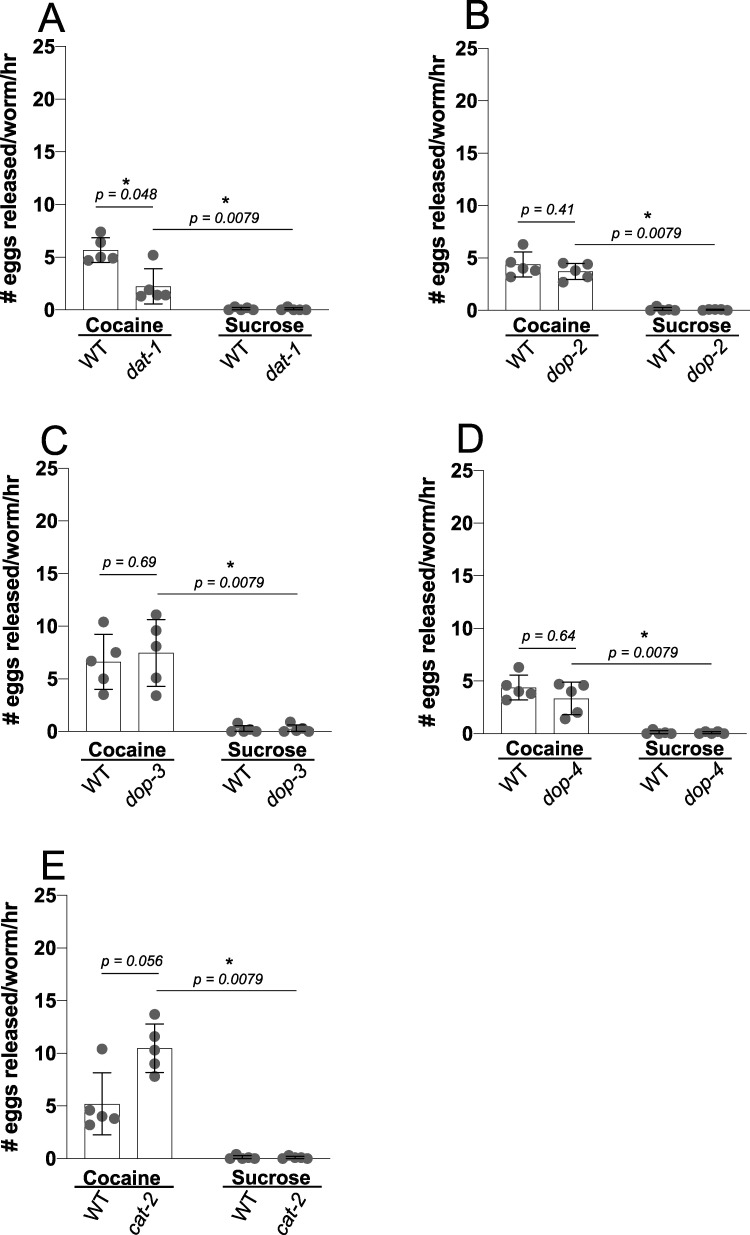
DAT is required for the egg laying response to cocaine, but not other DA signaling genes. Egg laying behavior of (A) DAT mutants *dat-1(ok157)*, (B) *dop-2(vs105)*, (C) *dop-3(vs106)*, (D) *dop-4(ok1321)*, and (E) *cat-2(e1112)* in 62.5 mM cocaine or a 62.5 mM sucrose control compared to WT. We performed five independent experiments with 10 animals in each group. Error bars represent SD. Mann–Whitney *U*-test. Significance indicated by asterisks.

The majority of mechanistic cocaine research has examined the drug’s activity as a DA reuptake inhibitor (Nestler 2005). According to this mechanism, cocaine binds to and blocks the DA reuptake transporter (DAT), preventing removal of DA from the synapse and thereby potentiating dopaminergic neurotransmission. *dat-1* encodes the *C. elegans* DAT, which has been shown to be sensitive to cocaine in heterologous systems (Jayanthi *et al.* 1998). To determine if DAT/*dat-1* is required for the egg laying response to cocaine in *C. elegans*, we tested the egg laying response to cocaine in DAT/*dat-1(ok157)* putative null mutants (McDonald *et al.* 2007). DAT/*dat-1(ok157)* putative null mutants exhibit a weak but significant decrease in the egg laying response to cocaine compared to WT [*dat-1(ok157)*, cocaine mean = 2.2 ± 1.7, WT, cocaine mean = 5.7 ± 1.2; *P *=* *0.048, *N* = 5 replicates (10 animals/replicate)] (Figure 9A). However, cocaine stimulates a significant increase in egg laying compared to equimolar sucrose control [*dat-1(ok157)*, cocaine mean = 2.2 ± 1.7; *dat-1(ok157)*, sucrose control mean = 0.2 ± 0.3; *P *=* *0.0079, *N* = 5 replicates (10 animals/replicate)].

Our analysis of the egg laying response to cocaine in mutants for the DA and 5-HT synthetic enzyme, AADC/*bas-1(ad446)* (Figure 1D), suggests that DA is not required for the egg laying response to cocaine. To further examine the role that DA plays in cocaine-stimulated egg laying, we tested the egg laying response to cocaine in a series of additional mutants with defects in DA signaling genes. The genotypes tested include putative null alleles of *dop-2(vs105)*, which encodes a Gα_o_-coupled DA receptor, *dop-3(vs106)*, which encodes a Gα_o_-coupled DA receptor, *dop-4(ok1321)*, which encodes a Gα_s_-coupled DA receptor, and *cat-2(e1112)*, which encodes the DA synthetic enzyme tyrosine hydroxylase (TH) (Lints and Emmons 1999; Chase *et al.* 2004; Cao and Aballay 2016; Fernandez *et al.* 2020). Consistent with our analysis of the egg laying response to cocaine in AADC/*bas-1(ad446)* mutants, neither TH/*cat-2(e1112)* mutants nor single mutants for the three genes encoding DA receptors expressed in the neurons and muscles that control *C. elegans* egg laying [*dop-2(vs105)*, *dop-3(vs106)*, and *dop-4(ok1321)*] exhibit a significant difference in the egg laying response to cocaine compared to WT (Figure 9, B–E). Together, these data suggest that DA is not a primary target of cocaine in this model system.

### Fraction of WT, cholinergic mutant, and monoamine mutant *C. elegans* that exhibit an egg laying response to cocaine

**Figure 10:**
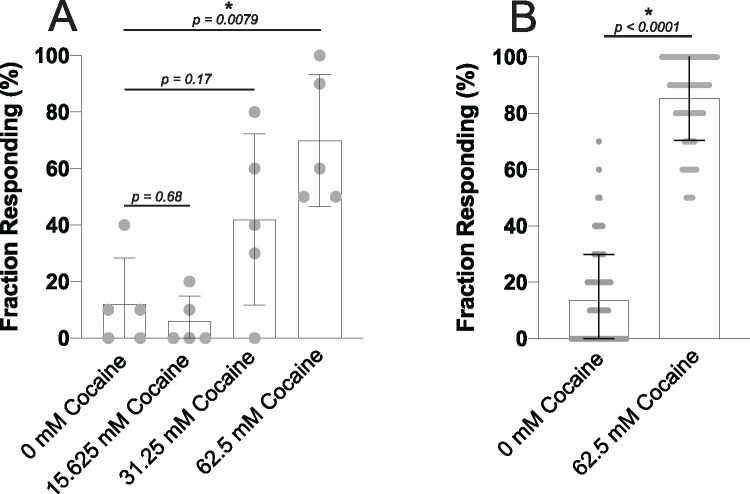
Fraction of WT *C. elegans* that emit an egg laying response to cocaine treatment. Fraction responding, defined as the fraction of animals releasing one or more eggs, of (A) WT *C. elegans* to a series of increasing concentrations of cocaine in liquid media and (B). WT *C. elegans* to 62.5 mM cocaine in liquid media compared to 62.5 mM sucrose control. For the data shown in (A), we performed five independent experiments with 10 animals in each group. For the data shown in panel (B), we re-analyzed 83 independent experiments with 10 animals in each group for which we had previously reported the mean number of eggs laid in 1 h. Error bars represent SD. Mann–Whitney *U*-test. Significance indicated by asterisks.

As is standard in the analysis of *C. elegans* egg laying behavior in response to pharmacological agents, we assayed the mean number of eggs laid in response to treatment by each of the genetic backgrounds tested compared to WT (Trent *et al.* 1983; Desai and Horvitz 1989; Schafer and Kenyon 1995; Weinshenker *et al.* 1995; Hart 2006). One limitation of comparing the mean eggs laid, however, is that it potentially obscures how the egg laying response to cocaine differs across the population of animals tested. To address this, we reanalyzed our data and calculated the fraction of WT animals laying at least one egg in response to treatment with cocaine in hypertonic media for 1 h (Figure 10, A and B). We show that cocaine stimulates an egg laying response in 85 ± 15% of WT animals tested whereas only 14 ± 16% of WT animals treated with equimolar sucrose control lay at least one egg [*P *<* *0.0001, *N* = 83 replicates (10 animals/replicate)] (Figure 10B).

We also reanalyzed the fraction responding to cocaine in all mutant strains tested compared to WT. The fraction of animals that lay at least one egg mostly followed the same pattern observed when analyzing the mean number of eggs laid in 1 h (Supplementary Tables S1 and S2).

## Discussion

### Cocaine may modulate endogenous cholinergic neurotransmission to stimulate *C. elegans* egg laying

In this study, we show that the mean egg laying response to cocaine in *C. elegans* is suppressed in mutants with defects in presynaptic cholinergic neurotransmission, postsynaptic defects in nAChRs, and defects in the *C. elegans* AChEs. Together, these results suggest that cocaine may modulate endogenous cholinergic neurotransmission to stimulate *C. elegans* egg laying through signaling at nAChRs. At this stage, the precise neurogenetic mechanism for the effects of cocaine on cholinergic neurotransmission remains unclear. Nonetheless, our novel findings demonstrate the utility of the *C. elegans* egg laying circuit as a model system for examination of the cholinergic effects of cocaine and lay the foundation for further mechanistic investigation.

A conclusive mechanism for the regulation of cholinergic neurotransmission by cocaine will require the collection of additional data. To this end, there are several mechanistic investigations that we suggest may shed light on the cholinergic effects of cocaine in future studies.

Cocaine may act on an uncharacterized receptor to regulate cholinergic neurotransmission. A recent biochemical report identified brain acid soluble protein 1 as a novel, pharmacologically relevant receptor for cocaine in mammals (Harraz *et al.* 2020). Brain acid soluble protein 1 is not conserved in *C. elegans*, but the identification of a novel cocaine receptor illustrates the possibility that cocaine may act on novel, noncanonical targets. Future studies could utilize the genetic tractability of *C. elegans* to identify additional noncanonical cocaine targets that regulate cholinergic neurotransmission via forward genetic screens.

In addition, cocaine may act simultaneously on one or more amine neurotransmitter systems or receptors to regulate cholinergic neurotransmission. *In vivo* micro dialysis studies in mammals have shown that by potentiating dopaminergic neurotransmission, cocaine increases ACh release from the nucleus accumbens and the hippocampus (Imperato *et al.* 1993; Mark *et al.* 1999). This raises the possibility that cocaine might act analogously through one or more amine neurotransmitters to regulate ACh release, possibly from the VCs, which are the primary cholinergic motor neurons in the *C*. *elegans* egg laying circuit or from the HSNs, which have also been reported to function as cholinergic motor neurons (Duerr *et al.* 2001; Schafer 2006; Pereira *et al.* 2015). Notably, none of the biogenic amine neurotransmitter mutants tested in this study besides DAT/*dat-1* mutants exhibit a suppressed mean egg laying response to cocaine compared to WT. However, because the egg laying circuit expresses multiple functionally redundant receptors for each of the biogenic amine neurotransmitters, it is possible that our genetic analysis of single and double mutants was insufficient to suppress possible amine-dependent egg laying responses to cocaine (Fernandez *et al.* 2020). Future studies could examine the egg laying behavior of triple and quadruple mutants for the receptors and synthetic enzymes of ACh and each of the amine neurotransmitter systems expressed in the egg laying circuit.

Notably, in a model where the egg laying response to cocaine is dependent on increased ACh signaling, the suppression of the egg laying response to cocaine in *ace-2*; *ace-1* double mutants is unexpected. Interestingly, mammals express two distinct types of cholinesterases, a “true” AChE with high substrate specificity for ACh and a less substrate-specific “pseudo” butrylcholinesterase that is the primary metabolic enzyme of cocaine in mammals (Gorelick 1997; Masson and Lockridge 2010). The substrate specificity of AChEs in *C. elegans* is intermediate to the distinct mammalian AChE and butrylcholinesterase (Johnson and Russell 1983; Kolson and Russell 1985). This raises the possibility that cocaine may be a substrate for *C. elegans* AChEs. Consequently, the suppression of the egg laying response to cocaine in these mutants may indicate that *C. elegans* AChEs metabolize cocaine to a compound which stimulates egg laying through a nicotinic cholinergic pathway. Alternatively, if *C. elegans* AChEs metabolize cocaine, this may inhibit the enzymes’ function as ACh-degrading enzymes, causing an increase in ACh levels at nAChRs. The role that the *C. elegans* AChEs play in the mode of action of cocaine is intriguing first, because it may be indicative of a possible novel mechanism of cocaine action. Second, interest in developing recombinant metabolic enzymes of cocaine and its associated metabolites as pharmacotherapeutic interventions for cocaine overdose and cocaine use disorder has accelerated over the last decade (Zheng and Zhan 2008; Zhang *et al.* 2017). Additional biochemical and genetic investigation of the effect of cocaine on *C. elegans* AChEs may therefore be a fruitful area of future investigation.

### The increase in cocaine-stimulated egg laying in SERT/*mod-5* and 5-HT-gated chloride channel/*mod-1* null animals may be 5-HT independent

Our data show that animals with null mutations in SERT/*mod-5* and the 5-HT-gated chloride channel/*mod-1* exhibit an enhanced cocaine-stimulated egg laying response compared to WT animals. These data could be interpreted as aligned with a model in which cocaine increases 5-HT levels, inducing egg laying in WT animals, an effect that is expected to be further potentiated by a lack of 5-HT reuptake in SERT/*mod-5* null animals. This serotonergic mechanism is supported by a study that investigated a cocaine-dependent decrease in locomotion speed in *C. elegans*, which is suppressed in 5-HT-gated chloride channel/*mod-1*, SERT/*mod-5*, and the 5-HT synthetic enzyme, TPH/*tph-1* null mutants (Ward *et al.* 2009).

However, we show that the null mutant in the 5-HT synthetic enzyme, TPH*/tph-1(mg280)* and the double mutant in the 5-HT receptors, *ser-7(tm1325) ser-1(ok345)*, do not suppress cocaine-stimulated egg laying, pointing to a 5-HT-independent mechanism in cocaine-stimulated egg laying. Additional data in SERT/*mod-5* and 5-HT signaling double mutants will be necessary to determine if the enhanced cocaine-stimulated egg laying observed in SERT/*mod-5* and 5-HT-gated chloride channel*/mod-1* null animals depends on endogenous 5-HT signaling.

Interestingly, recent reports using calcium imaging of the egg laying circuit in freely behaving worms show evidence of coordination between egg laying behavior, body bend, and locomotion (Zhang *et al.* 2008; Collins and Koelle 2013; Collins *et al.* 2016; Kopchock *et al.* 2021). Such detailed analysis of the coordinated function of both circuits in cocaine-treated WT and mutant animals *in vivo* will be highly valuable to directly describe the neurobiological mechanism of cocaine action in *C. elegans*.

### Potential of the egg laying circuit as a model system for the cholinergic effects of cocaine

In this study, we show that, except for a putative null allele of DAT/*dat-1*, mutants with defects in the amine neurotransmitters DA, Tyr, Oct, and 5-HT do not exhibit a suppressed mean egg laying response to cocaine compared to WT. This is a surprising finding because cocaine’s canonical mechanism of action is potentiation of monoaminergic neurotransmission through blockade of monoamine reuptake transporters (Richards and Laurin 2020). We do, however, show that cocaine-stimulated egg laying is suppressed in animals with defects in nicotinic cholinergic neurotransmission, which suggests that cocaine may modulate endogenous ACh signaling to induce egg laying through stimulation of nAChRs on the vulval muscles. This possibility aligns with previous reports that cocaine increases ACh release in multiple structures in the limbic system (Imperato *et al.* 1993; Mark *et al.* 1999). At present, the mechanism underlying potential modulation of cholinergic neurotransmission by cocaine is unclear. Nonetheless, the results of this study provide a background for future investigation utilizing the *C. elegans* egg laying circuit to dissect the detailed molecular mechanisms underlying the potential conserved cholinergic effects of cocaine.

## References

[jkab143-B1] Alfonso A , Grundahl K , Duerr JS , Han HP , Rand JB. 1993. The *Caenorhabditis elegans* unc-17 gene: a putative vesicular acetylcholine transporter. Science. 261:617–619. 8342028 10.1126/science.8342028

[jkab143-B2] Alkema MJ , Hunter-Ensor M , Ringstad N , Horvitz HR. 2005. Tyramine functions independently of octopamine in the *Caenorhabditis elegans* nervous system. Neuron. 46:247–260. 15848803 10.1016/j.neuron.2005.02.024

[jkab143-B3] Arpagaus M , Combes D , Culetto E , Grauso M , Fedon Y , et al 1998. Four acetylcholinesterase genes in the nematode *Caenorhabditis elegans*. J Physiol Paris. 92:363–367. 9789838 10.1016/S0928-4257(99)80006-0

[jkab143-B4] Bany IA , Dong M-Q , Koelle MR. 2003. Genetic and cellular basis for acetylcholine inhibition of *Caenorhabditis elegans* egg-laying behavior. J Neurosci. 23:8060–8069. 12954868 10.1523/JNEUROSCI.23-22-08060.2003 PMC6740490

[jkab143-B5] Cao X , Aballay A. 2016. Neural inhibition of dopaminergic signaling enhances immunity in a cell non-autonomous manner. Curr Biol. 26:2329–2334. 27524480 10.1016/j.cub.2016.06.036 PMC5021569

[jkab143-B6] Carnell L , Illi J , Hong SW , McIntire SL. 2005. The G-Protein-coupled serotonin receptor SER-1 regulates egg laying and male mating behaviors in *Caenorhabditis elegans*. J Neurosci. 25:10671–10681. 16291940 10.1523/JNEUROSCI.3399-05.2005 PMC6725853

[jkab143-B7] Chase D. 2007. Biogenic Amine Neurotransmitters in C. elegans. WormBook. doi:10.1895/wormbook.1.132.1. (Accessed: 2020 April 15). http://www.wormbook.org/chapters/www_monoamines/monoamines.html. 10.1895/wormbook.1.132.1 PMC4781333 18050501

[jkab143-B8] Chase DL , Pepper JS , Koelle MR. 2004. Mechanism of extrasynaptic dopamine signaling in *Caenorhabditis elegans*. Nat Neurosci. 7:1096–1103. 15378064 10.1038/nn1316

[jkab143-B9] Churgin MA , McCloskey RJ , Peters E , Fang-Yen C. 2017. Antagonistic serotonergic and octopaminergic neural circuits mediate food-dependent locomotory behavior in *Caenorhabditis elegans*. J Neurosci. 37:7811–7823. 28698386 10.1523/JNEUROSCI.2636-16.2017 PMC5559759

[jkab143-B10] Collins KM , Bode A , Fernandez RW , Tanis JE , Brewer JC , et al 2016. Activity of the *C. elegans* egg-laying behavior circuit is controlled by competing activation and feedback inhibition. eLife. 10.7554/eLife.21126 PMC5142809 27849154

[jkab143-B11] Collins KM , Koelle MR. 2013. Postsynaptic ERG potassium channels limit muscle excitability to allow distinct egg-laying behavior states in *Caenorhabditis elegans*. J Neurosci. 33:761–775. 23303953 10.1523/JNEUROSCI.3896-12.2013 PMC3542984

[jkab143-B12] Combes D , Fedon Y , Grauso M , Toutant JP , Arpagaus M. 2000. Four genes encode acetylcholinesterases in the nematodes *Caenorhabditis elegans* and *Caenorhabditis briggsae*. cDNA sequences, genomic structures, mutations and *in vivo* expression. J Mol Biol. 300:727–742. 10891266 10.1006/jmbi.2000.3917

[jkab143-B13] Combes D , Fedon Y , Toutant JP , Arpagaus M. 2001. Acetylcholinesterase genes in the nematode *Caenorhabditis elegans*. Int Rev Cytol. 209:207–239. 11580201 10.1016/s0074-7696(01)09013-1

[jkab143-B14] Conradt B , Horvitz HR. 1998. The *C. elegans* protein EGL-1 is required for programmed cell death and interacts with the Bcl-2–like Protein CED-9. Cell. 93:519–529. 9604928 10.1016/s0092-8674(00)81182-4

[jkab143-B15] Culotti JG , Von Ehrenstein G , Culotti MR , Russell RL. 1981. A second class of acetylcholinesterase-deficient mutants of the nematode *Caenorhabditis elegans*. Genetics. 97:281–305. 7274655 10.1093/genetics/97.2.281 PMC1214394

[jkab143-B16] Czoty PW , Stoops WW , Rush CR. 2016. Evaluation of the “pipeline” for development of medications for cocaine use disorder: a review of translational preclinical, human laboratory, and clinical trial research. Pharmacol Rev. 68:533–562. 27255266 10.1124/pr.115.011668 PMC4931869

[jkab143-B17] Dempsey CM , Mackenzie SM , Gargus A , Blanco G , Sze JY. 2005. Serotonin (5HT), Fluoxetine, imipramine and dopamine target distinct 5HT receptor signaling to modulate *Caenorhabditis elegans* egg-laying behavior. Genetics. 169:1425–1436. 15654117 10.1534/genetics.104.032540 PMC1449529

[jkab143-B18] Desai C , Horvitz HR. 1989. *Caenorhabditis elegans* mutants defective in the functioning of the motor neurons responsible for egg laying. Genetics. 121:703–721. 2721931 10.1093/genetics/121.4.703 PMC1203655

[jkab143-B19] Duerr JS , Gaskin J , Rand JB. 2001. Identified neurons in *C. elegans* coexpress vesicular transporters for acetylcholine and monoamines. Am J Physiol Cell Physiol. 280:C1616–C1622. 11350757 10.1152/ajpcell.2001.280.6.C1616

[jkab143-B20] Dvir H , Silman I , Harel M , Rosenberry TL , Sussman JL. 2010. Acetylcholinesterase: from 3D structure to function. Chem Biol Interact. 187:10–22. 20138030 10.1016/j.cbi.2010.01.042 PMC2894301

[jkab143-B21] Engleman EA , Katner SN , Neal-Beliveau BS. 2016. *Caenorhabditis elegans* as a model to study the molecular and genetic mechanisms of drug addiction. Prog Mol Biol Transl Sci. 137:229–252. 26810004 10.1016/bs.pmbts.2015.10.019 PMC4834862

[jkab143-B22] Engleman EA , Steagall KBI , Bredhold KE , Breach M , Kline HL , et al 2018. *Caenorhabditis elegans* show preference for stimulants and potential as a model organism for medications screening. Front Physiol. 9:1200. 30214414 10.3389/fphys.2018.01200 PMC6125605

[jkab143-B23] Fernandez RW , Wei K , Wang EY , Mikalauskaite D , Olson A , et al 2020. Cellular expression and functional roles of all 26 neurotransmitter GPCRs in the *C. elegans* egg-laying circuit. J Neurosci. 40:7475–7488. 32847964 10.1523/JNEUROSCI.1357-20.2020 PMC7511189

[jkab143-B24] Fleming JT , Squire MD , Barnes TM , Tornoe C , Matsuda K , Ahnn J , et al 1997. *Caenorhabditis elegans* levamisole resistance geneslev-1, unc-29, and unc-38 encode functional nicotinic acetylcholine receptor subunits. J Neurosci. 17:5843–5857. 9221782 10.1523/JNEUROSCI.17-15-05843.1997 PMC6573193

[jkab143-B25] Gorelick DA. 1997. Enhancing cocaine metabolism with butyrylcholinesterase as a treatment strategy. Drug Alcohol Depend. 48:159–165. 9449014 10.1016/s0376-8716(97)00119-1

[jkab143-B26] Gottschling D-C , Döring F , Lüersen K. 2017. Locomotion behavior is affected by the GαS pathway and the two-pore-domain K+ channel TWK-7 interacting in GABAergic motor neurons in *Caenorhabditis elegans*. Genetics. 206:283–297. 28341653 10.1534/genetics.116.195669 PMC5419475

[jkab143-B27] Hapiak VM , Hobson RJ , Hughes L , Smith K , Harris G , et al 2009. Dual excitatory and inhibitory serotonergic inputs modulate egg laying in *Caenorhabditis elegans*. Genetics. 181:153–163. 19001289 10.1534/genetics.108.096891 PMC2621164

[jkab143-B28] Hare EE , Loer CM. 2004. Function and evolution of the serotonin-synthetic bas-1 gene and other aromatic amino acid decarboxylase genes in Caenorhabditis. BMC Evol Biol. 4:24. 15287963 10.1186/1471-2148-4-24 PMC514703

[jkab143-B29] Harraz MM , Malla AP , Semenza ER , Shishikura M , Hwang Y , et al 2020. Cocaine receptor identified as BASP1. bioRxiv. 2020.11.23.392787.

[jkab143-B30] Hart A. 2006. Behavior. WormBook. doi:10.1895/wormbook.1.87.1. (Accessed: 2020 October 19). http://www.wormbook.org/chapters/www_behavior/behavior.html.

[jkab143-B31] Hasselmo ME. 2006. The role of acetylcholine in learning and memory. Curr Opin Neurobiol. 16:710–715. 17011181 10.1016/j.conb.2006.09.002 PMC2659740

[jkab143-B32] Hedegaard H , Spencer MR , Garnett M. 2020. Increase in drug overdose deaths involving cocaine: United States 2009–2018. NCHS Data Brief No. 384. 33054918

[jkab143-B33] Higgins GA , Fletcher PJ. 2003. Serotonin and drug reward: focus on 5-HT2C receptors. Eur J Pharmacol. 480:151–162. 14623358 10.1016/j.ejphar.2003.08.102

[jkab143-B34] Hobson RJ , Hapiak VM , Xiao H , Buehrer KL , Komuniecki PR , et al 2006. SER-7, a *Caenorhabditis elegans* 5-HT7-like receptor, is essential for the 5-HT stimulation of pharyngeal pumping and egg laying. Genetics. 172:159–169. 16204223 10.1534/genetics.105.044495 PMC1456143

[jkab143-B35] Horvitz HR , Chalfie M , Trent C , Sulston JE , Evans PD. 1982. Serotonin and octopamine in the nematode *Caenorhabditis elegans*. Science. 216:1012–1014. 6805073 10.1126/science.6805073

[jkab143-B36] Imperato A , Obinu MC , Gessa GL. 1993. Effects of cocaine and amphetamine on acetylcholine release in the hippocampus and caudate nucleus. Eur J Pharmacol. 238:377–381. 8405105 10.1016/0014-2999(93)90869-j

[jkab143-B37] Jayanthi LD , Apparsundaram S , Malone MD , Ward E , Miller DM , et al 1998. The *Caenorhabditis elegans* gene T23G5.5 encodes an antidepressant- and cocaine-sensitive dopamine transporter. Mol Pharmacol. 54:601–609. 9765501

[jkab143-B38] Johnson CD , Russell RL. 1983. Multiple Molecular Forms of Acetylcholinesterase in the Nematode *Caenorhabditis elegans*. J Neurochem. 41:30–46. 6864228 10.1111/j.1471-4159.1983.tb11811.x

[jkab143-B39] Kampman KM. 2019. The treatment of cocaine use disorder. Sci Adv. 5:eaax1532. 31663022 10.1126/sciadv.aax1532 PMC6795516

[jkab143-B40] Kim J , Poole DS , Waggoner LE , Kempf A , Ramirez DS , et al 2001. Genes affecting the activity of nicotinic receptors involved in *Caenorhabditis elegans* egg-laying behavior. Genetics. 157:1599–1610. 11290716 10.1093/genetics/157.4.1599 PMC1461590

[jkab143-B41] Klinkenberg I , Sambeth A , Blokland A. 2011. Acetylcholine and attention. Behav Brain Res. 221:430–442. 21108972 10.1016/j.bbr.2010.11.033

[jkab143-B42] Kolson DL , Russell RL. 1985. A novel class of acetylcholinesterase, revealed by mutations, in the nematode *Caenorhabditis elegans*. J Neurogenet. 2:93–110. 4020535 10.3109/01677068509100145

[jkab143-B43] Kopchock RJ , Ravi B , Bode A , Collins KM. 2021. The sex-specific VC neurons are mechanically activated motor neurons that facilitate serotonin-induced egg laying in *C. elegans*. J Neurosci. doi:10.1523/JNEUROSCI.2150-20.2021 10.1523/JNEUROSCI.2150-20.2021 PMC8055074 33687965

[jkab143-B44] Lackner MR , Nurrish SJ , Kaplan JM. 1999. Facilitation of synaptic transmission by EGL-30 Gqα and EGL-8 PLCβ: DAG Binding to UNC-13 is required to stimulate acetylcholine release. Neuron. 24:335–346. 10571228 10.1016/s0896-6273(00)80848-x

[jkab143-B45] Lints R , Emmons SW. 1999. Patterning of dopaminergic neurotransmitter identity among *Caenorhabditis elegans* ray sensory neurons by a TGFbeta family signaling pathway and a Hox gene. Dev Camb Engl. 126:5819–5831. 10.1242/dev.126.24.5819 10572056

[jkab143-B46] Lipari RN , Van Horn SL. 2013. Trends in Substance Use Disorders Among Adults Aged 18 or Older. Rockville, MD: Substance Abuse and Mental Health Services Administration (US). (Accessed: 2020 September 1). http://www.ncbi.nlm.nih.gov/books/NBK447253/. 28792721

[jkab143-B47] Liu Y , LeBoeuf B , Garcia LR. 2007. Gαq-coupled muscarinic acetylcholine receptors enhance nicotinic acetylcholine receptor signaling in *Caenorhabditis elegans* mating behavior. J Neurosci. 27:1411–1421. 17287516 10.1523/JNEUROSCI.4320-06.2007 PMC6673585

[jkab143-B48] Mark GP , Hajnal A , Kinney AE , Keys AS. 1999. Self-administration of cocaine increases the release of acetylcholine to a greater extent than response-independent cocaine in the nucleus accumbens of rats. Psychopharmacology (Berl). 143:47–53. 10227079 10.1007/s002130050918

[jkab143-B49] Masson P , Lockridge O. 2010. Butyrylcholinesterase for protection from organophosphorus poisons; catalytic complexities and hysteretic behavior. Arch Biochem Biophys. 494:107. 20004171 10.1016/j.abb.2009.12.005 PMC2819560

[jkab143-B50] McDonald PW , Hardie SL , Jessen TN , Carvelli L , Matthies DS , et al 2007. Vigorous motor activity in *Caenorhabditis elegans* requires efficient clearance of dopamine mediated by synaptic localization of the dopamine transporter DAT-1. J Neurosci. 27:14216–14227. 18094261 10.1523/JNEUROSCI.2992-07.2007 PMC6673513

[jkab143-B51] Müller CP , Carey RJ , Huston JP , De Souza Silva MA. 2007. Serotonin and psychostimulant addiction: focus on 5-HT1A-receptors. Prog Neurobiol. 81:133–178. 17316955 10.1016/j.pneurobio.2007.01.001

[jkab143-B52] Nestler EJ. 2005. The neurobiology of cocaine addiction. Sci Pract Perspect. 3:4–10. 18552739 10.1151/spp05314 PMC2851032

[jkab143-B53] Olde B , McCombie WR. 1997. Molecular cloning and functional expression of a serotonin receptor from *Caenorhabditis elegans*. J Mol Neurosci. 8:53–62. 9061615 10.1007/BF02736863

[jkab143-B54] Pereira L , Kratsios P , Serrano-Saiz E , Sheftel H , Mayo AE , et al 2015. A cellular and regulatory map of the cholinergic nervous system of *C. elegans*. eLife. 4:e12432. 26705699 10.7554/eLife.12432 PMC4769160

[jkab143-B55] Pitts DK , Marwah J. 1989. Autonomic actions of cocaine. Can J Physiol Pharmacol. 67:1168–1176. 2688865 10.1139/y89-186

[jkab143-B56] Rand J. 2007. Acetylcholine. WormBook. doi:10.1895/wormbook.1.131.1. (Accessed: 2020 April 15). http://www.wormbook.org/chapters/www_acetylcholine/acetylcholine.html.

[jkab143-B57] Rand JB , Russell RL. 1984. Choline acetyltransferase-deficient mutants of the nematode *Caenorhabditis elegans*. Genetics. 106:227–248. 6698395 10.1093/genetics/106.2.227 PMC1202253

[jkab143-B58] Rand JB , Russell RL. 1985. Properties and partial purification of choline acetyltransferase from the nematode *Caenorhabditis elegans*. J Neurochem. 44:189–200. 3964827 10.1111/j.1471-4159.1985.tb07130.x

[jkab143-B59] Ranganathan R , Cannon SC , Horvitz HR. 2000. MOD-1 is a serotonin-gated chloride channel that modulates locomotory behaviour in *C. elegans*. Nature. 408:470–475. 11100728 10.1038/35044083

[jkab143-B60] Ranganathan R , Sawin ER , Trent C , Horvitz HR. 2001. Mutations in the *Caenorhabditis elegans* serotonin reuptake transporter MOD-5 reveal serotonin-dependent and -independent activities of fluoxetine. J Neurosci. 21:5871–5884. 11487610 10.1523/JNEUROSCI.21-16-05871.2001 PMC6763176

[jkab143-B61] Richards JR , Laurin EG. 2020. StatPearls. In: Cocaine. Treasure Island, FL: StatPearls Publishing. (Accessed: 2020 June 24). http://www.ncbi.nlm.nih.gov/books/NBK430769/.

[jkab143-B62] Richmond JE , Jorgensen EM. 1999. One GABA and two acetylcholine receptors function at the *C. elegans* neuromuscular junction. Nat Neurosci. 2:791–797. 10461217 10.1038/12160 PMC2585773

[jkab143-B63] Schafer W. 2005. Egg-laying. WormBook. doi:10.1895/wormbook.1.38.1. (Accessed: 2019 August 19). http://www.wormbook.org/chapters/www_egglaying/egglaying.html

[jkab143-B64] Schafer WR. 2006. Genetics of egg-laying in worms. Annu Rev Genet. 40:487–509. 17094742 10.1146/annurev.genet.40.110405.090527

[jkab143-B65] Schafer WR , Kenyon CJ. 1995. A calcium-channel homologue required for adaptation to dopamine and serotonin in *Caenorhabditis elegans*. Nature. 375:73–78. 7723846 10.1038/375073a0

[jkab143-B66] Sofuoglu M , Mooney M. 2009. Cholinergic functioning in stimulant addiction: implications for medications development. CNS Drugs. 23:939–952. 19845415 10.2165/11310920-000000000-00000 PMC2778856

[jkab143-B67] Stiernagle T. 2006. Maintenance of *C. elegans*. WormBook. doi:10.1895/wormbook.1.101.1. (Accessed: 2020 July 30). http://www.wormbook.org/chapters/www_strainmaintain/strainmaintain.html 10.1895/wormbook.1.101.1 PMC4781397 18050451

[jkab143-B68] Sze JY , Victor M , Loer C , Shi Y , Ruvkun G. 2000. Food and metabolic signalling defects in a *Caenorhabditis elegans* serotonin-synthesis mutant. Nature. 403:560–564. 10676966 10.1038/35000609

[jkab143-B69] The C. elegans Deletion Mutant Consortium 2012. Large-scale screening for targeted knockouts in the *Caenorhabditis elegans*. Genome. G3 (Bethesda). 2:1415–1425. 23173093 10.1534/g3.112.003830 PMC3484672

[jkab143-B70] Trent C , Tsung N , Horvitz HR. 1983. Egg-laying defective mutants of the nematode *Caenorhabditis elegans*. Genetics. 104:619–647. 11813735 10.1093/genetics/104.4.619 PMC1202130

[jkab143-B71] Ward A , Walker VJ , Feng Z , Xu XZS. 2009. Cocaine modulates locomotion behavior in *C. elegans*. PLoS One. 4:e5946. 19536276 10.1371/journal.pone.0005946 PMC2691951

[jkab143-B72] Weinshenker D , Garriga G , Thomas JH. 1995. Genetic and pharmacological analysis of neurotransmitters controlling egg laying in *C. elegans*. J Neurosci. 15:6975–6985. 7472454 10.1523/JNEUROSCI.15-10-06975.1995 PMC6577982

[jkab143-B73] White JG , Southgate E , Thomson JN , Brenner S. 1986. The structure of the nervous system of the nematode *Caenorhabditis elegans*. Philos Trans R Soc Lond B Biol Sci. 314:1–340. 22462104 10.1098/rstb.1986.0056

[jkab143-B74] Zhang M , Chung SH , Fang-Yen C , Craig C , Kerr RA , et al 2008. A self-regulating feed-forward circuit controlling *C. elegans* egg-laying behavior. Curr Biol. 18:1445–1455. 18818084 10.1016/j.cub.2008.08.047 PMC2621019

[jkab143-B75] Zhang T , Zheng X , Zhou Z , Chen X , Jin Z , et al 2017. Clinical potential of an enzyme-based novel therapy for cocaine overdose. Sci Rep. 7:1–9. 29127295 10.1038/s41598-017-14105-5 PMC5681513

[jkab143-B76] Zheng F , Zhan C-G. 2008. Rational design of an enzyme mutant for anti-cocaine therapeutics. J Comput Aided Mol Des. 22:661–671. 17989928 10.1007/s10822-007-9144-9 PMC2875689

